# Paddle Stroke Analysis for Kayakers Using Wearable Technologies

**DOI:** 10.3390/s21030914

**Published:** 2021-01-29

**Authors:** Long Liu, Hui-Hui Wang, Sen Qiu, Yun-Cui Zhang, Zheng-Dong Hao

**Affiliations:** 1 Dalian Neusoft University of Information, Dalian 116023, China; liulong@neusoft.edu.cn (L.L.); wanghuihui@neusoft.edu.cn (H.-H.W.); 2 The Laboratory of Intelligent System, Dalian University of Technology, Dalian 116024, China; bayin@mail.dlut.edu.cn; 3 The Research Institute of Photonics, Dalian Polytechnic University, Dalian 116023, China; zhang_yc@dlpu.edu.cn

**Keywords:** paddle stroke analysis, motion reconstruction, inertial sensor, data fusion

## Abstract

Proper stroke posture and rhythm are crucial for kayakers to achieve perfect performance and avoid the occurrence of sport injuries. The traditional video-based analysis method has numerous limitations (e.g., site and occlusion). In this study, we propose a systematic approach for evaluating the training performance of kayakers based on the multiple sensors fusion technology. Kayakers’ motion information is collected by miniature inertial sensor nodes attached on the body. The extend Kalman filter (EKF) method is used for data fusion and updating human posture. After sensor calibration, the kayakers’ actions are reconstructed by rigid-body model. The quantitative kinematic analysis is carried out based on joint angles. Machine learning algorithms are used for differentiating the stroke cycle into different phases, including entry, pull, exit and recovery. The experiment shows that our method can provide comprehensive motion evaluation information under real on-water scenario, and the phase identification of kayaker’s motions is up to 98% validated by videography method. The proposed approach can provide quantitative information for coaches and athletes, which can be used to improve the training effects.

## 1. Introduction

As a multi-cycle high-intensity water sports project, kayak includes single boat, double boat, four-person boat and obstacle slalom formats. The athlete sits in the boat, facing forward, holding the paddle with oar handle on the middle position, relying on the feet to steer the rudder to control the course. kayaking is closely related to athletes’ professional skills, physical fitness, psychological state and other aspects, and the key to win in the competition is to complete the paddling movement efficiently and without any mistakes under tense conditions. Professional teams and amateur clubs are constantly looking for advanced methods to help athletes improve their athletic performance [1,2,3].

Several different methods have been used for rowing technique testing. Video-based analysis by researchers to quantify the stroke performance of rowers is one of them [4], but this approach is restricted by the experimental site, which suffers from visual blind field, and it does not observe the behavior accurately. Other studies have been devoted to the creation of instrumented boats to assess rowers’ performance by measuring oar’s power and motion [5]. Franz Gravenhorst et al. assessed rowing technology by continuously monitoring rowers’ seat positions [6]. Henry et al. used strain gauges and potentiometers to measure the forces on the oars and their angular positions to assess rowing performance by power output [7]. Although boat speed, stroke frequency, stroke force and power output of athletes are evaluated, the standardization and normalization of rowing motion are not studied to fundamentally improve the kayaker’s technique.

All the above studies are to test kayaking equipment by instruments, so as to study the rowing performance. However, kayaking is a cooperative movement of athletes’ arms, torso, upper limbs and body along a certain movement track, which is a combination of factors of athletes’ muscle activity, joint flexion/extension angle and limbs activity [8,9]. Therefore, wearable sensors can evaluate the skills of rowing sports based on athletes’ movements capture. At present, it is a new trend to use inertial sensors on evaluating rowing performance [10,11]. M.Tesconi et al. developed a tight wearable sensor system, but it is only tested in the laboratory without extensive practical testing. In fact, the effort of balance control in on-water scenario results in clumsiness and change in the motor part of the action, and further leads to discredit on the simulated indoor experiments [12]. Rachel C. King et al. introduced a kinematic monitoring system which combines inertial sensors and other body sensor network nodes. However, the rotation of the back and femur in the sagittal plane is mainly monitored, and the flexion and extension of the upper limbs are not studied. The rowing action consists of shoulder abduction/adduction and elbow flexion/extension, and a lack of analysis in coronal plane is worth consideration [13]. Ruffaldi et al. put forward a sensor fusion model which integrates wearable inertial measurement with physiological sensors and marks the buttocks, sternum, acromion, outside humerus, medial epicondyle, ulna and radial styloid process. This method can support the human motion tracking of rowing in indoor and outdoor environments. However, the experimental results and discussions are defined within the indoor training, and the absence of real rowing data is a major barrier for true evaluation [14]. Taken together, the quantitative analysis of kayaking athletes’ movement in the above research is relatively incomplete, and there is limited research on monitoring and analyzing athletes’ whole-body movement [15].

To improve athletes’ rowing skills and provide the comprehensive technical guidance of kayaking sprint, we put forward a method of motion reconstruction and analysis based on inertial measurement units (IMUs). In our study, the athlete body is regarded as a set of rigid models, including several segments with self-defined length, and each body segment is modeled as a line which is connected by the friction-free joints. The attitude information is described by means of quaternions. Based on the quaternion-driven rotation, the joint angle of flexion and extension movement of each human body part is fully described.

The main contributions of this work are as follows:

- We use extend Kalman filter to fuse inertial sensor data and reconstruct real-time postures of kayakers in different paddling positions and capture the movements of single kayakers under realistic conditions.
- The validity and accuracy of the proposed posture estimation algorithm is verified using an optical motion capture system.
- Stroke quality of single kayaker is analyzed based on joint angles obtained from motion reconstruction.
- We use machine learning algorithms for phase partitioning of a stroke cycle.

The article is structured as follows. Section 2 introduces the hardware and software platform. The experimental methodology is described in Section 3. The results of this study is described are Section 4. Finally, discussions and conclusions are given in Section 5 and Section 6, respectively.

## 2. Systematic Data Collection and Participants

### 2.1. System Platform

**Figure 1:**
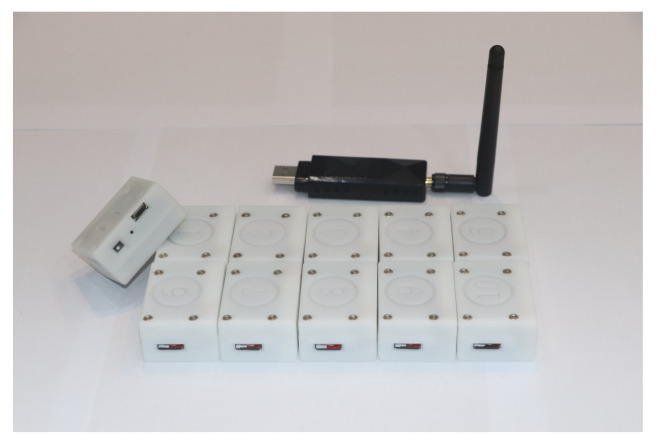
Diagram of apparatus components.

**Figure 2:**
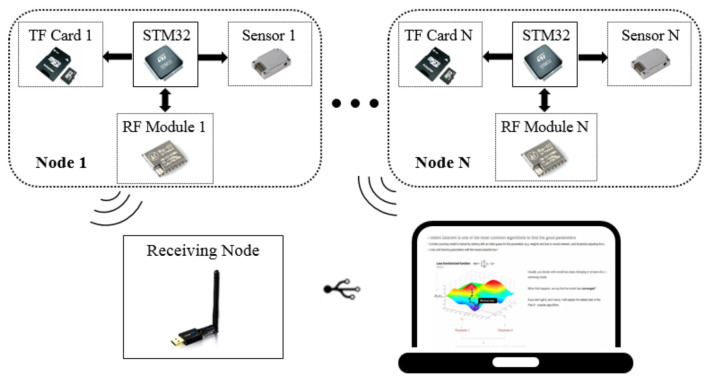
Schematic illustration of the self-designed motion capture system structure.

The IMUs-based motion tracking system used to obtain attitude of rowers was self-designed in our lab. The total system consists of several tiny inertial measurement units, a transceiver and a set of self-designed software. The physical device is shown in Figure 1. Each inertial measurement unit contains a triaxial accelerometer, a triaxial gyroscope and a triaxial magnetometer to measure the three-shaft acceleration, three-shaft angular velocity, and three-shaft magnetic field intensity. The detailed specifications are shown in Table 1. We used an STM32 as the micro control unit to record information from the inertial sensor units and store the raw data in a TransFlash card. The motion captured process by IMUs is controlled wirelessly by the Lora signal sent by the computer. In every sensor node, there is a miniature Lora wireless module soldered to the printed circuit board of the slave node. The main controller communicates with the RF module via SPI interface, and it is always waiting for an interrupt to start acquisition of inertial measurement. Once the master transceiver connected to the computer by USB interface receives the “START” signal from the self-made software, it delivers in the broadcasting signal. Upon receiving the public signal, the slave receivers start to collect data simultaneously. In addition, after the initial stage is completed, the participants are required to perform specific movements (stomp and shake hands) for synchronization of different sensor nodes. The system acquires the raw data at a sampling rate of 400 Hz. When the operation of the capture process is completed, the data stored in the memory card are exported for subsequent kinematic data analysis. A 3.7 V (400 mAh) battery is selected to power the whole system. At the end of each experiment, the collected data are immediately copied to the personal computer. The apparatus is preceded by practice trials, and the test battery lasted approximately 2 h. Each experiment period is about 10 min per volunteer. The composition of motion capture system is depicted in Figure 2.

### 2.2. Participants and Experimental Sites

Six kayakers recruited from the provincial sprint team participates in the preliminary study. The training duration for each participant is approximately more that three years. The participants train six times per week, with daily training sessions of 5–6 h. They have an average weight of 72.4 ± 6.4 kg and height of 1.76 ± 0.33 m. They are all female athletes. All participants had their height and weight recorded, and they were fully informed and consent was obtained. The experiment was conducted at the Sports Training Center in Dalian, Liaoning Province, China (latitude 121°15.194′ N and longitude 38°55.467′ E).

## 3. Methods

### 3.1. Rigid Body Model

**Figure 3:**
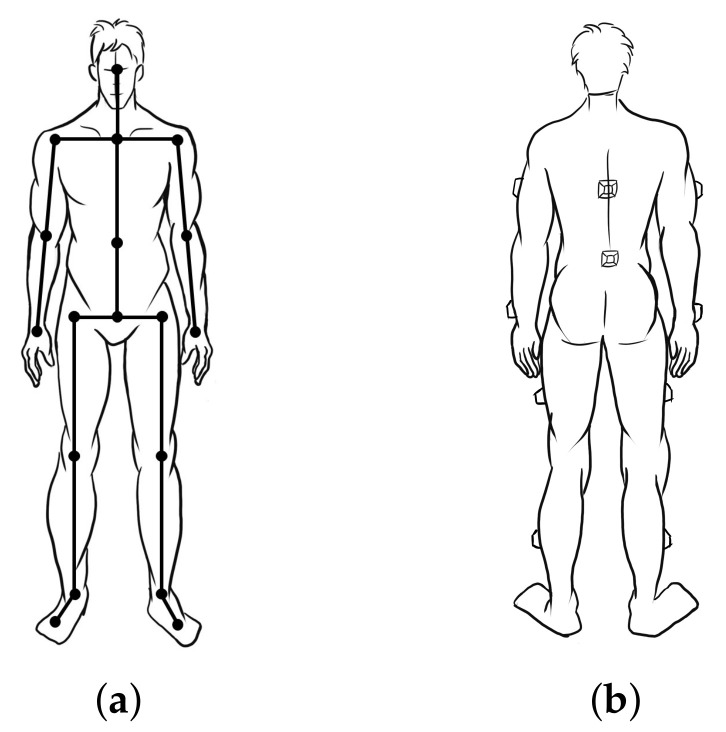
Schematic of the whole body structure definition with rigid body model and the distributed representation of the sensor’s location: (**a**) a rigid-body model with 17 human body segments connected via revolute joints, and the segment number can be adjusted according to the specific situation; and (**b**) the location and method of fixation for the sensors.

In the kinematic analysis of this paper, the body is defined as a rigid structure, and the skeletal structural model of the rower can be defined by 17 rigid segments (feet, calves, thighs, pelvis, waist, chest, arms, thighs and head), as shown in Figure 3a. The kayaker’s pelvis is set as the zero point. The length of each segment is approximately proportionally determined by the height of the athlete. As the line segments are connected by joint, if the orientation described by Euler angle or quaternion of each segment is obtained, the positions of the other lines can be determined by the length of the skeletal vector in the attitude in Figure 3a by iterative calculation [16]. To capture the limbs kinematical information, the nine-axis inertial measurement is placed on each of the back, waist, thigh, arm, calf and the limb segments, which is used to obtain raw acceleration, angular velocity and magnetometer information during the acquisition process. The placements of inertial sensors are shown in Figure 3b. The collection of kinematic data from head and feet was not the focus of this research, and the sensor nodes for head and feet motion capture were removed. The solutions of motion reconstruction data are replaced by the neighboring nodes. Thus, capturing full-body human motion needs 10 sensor nodes, as depicted in Figure 3b, and only six sensor nodes are required for capturing upper limbs.

**Figure 4:**
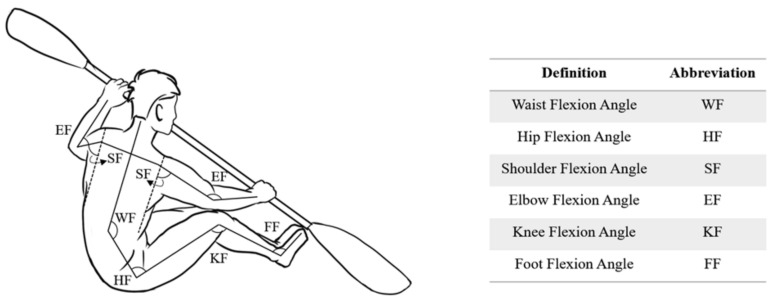
The definition of body joint angle.

The joint angles are defined as the angle between the vectors connecting adjacent body segments. The changes in posture of kayakers corresponds to the flexion and extension angle of each joint. The joint angles are depicted in Figure 4. The kayaking movement referred to the athlete who sits in the kayak and the paddling movement is primarily achieved by the upper limbs, so the flexion/extension of the shoulder joint (SF) and flexion/extension of the elbow joint (EF) are the main aspects of our approach [17].

### 3.2. Precondition of Rehabilitation Motion Design

**Figure 5:**
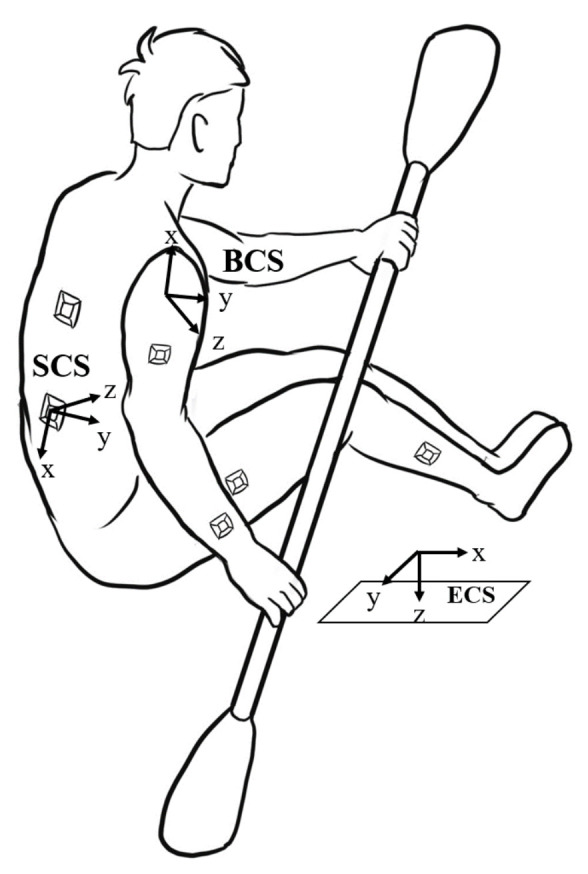
Definition of the coordinate systems used in this work, and including Earth Coordinate System (ECS), Body Coordinate System (BCS) and Sensor Coordinate System (SCS).

As shown in Figure 5, the whole motion capture system contains three coordinate systems, and each three-dimensional coordinate system is based on the standard right-handed 3D cartesian coordinate system. During the experiment, the inertial sensors are secured to each body segment by Velcro straps to acquire information related to the bodily motions of the participants.

The sensor returns signal in local coordinates, which is called Sensor Coordinate System (SCS). It is defined as the coordinates of a sensor node placed on the human body. However, the motion is observed in the Earth Coordinate System (ECS). The trajectories of each body segment and joint are measured with respect to the ECS, as opposed to the Body Coordinate System (BCS). Therefore, the raw inertial data time series are each transformed from SCS to ECS using the rotation matrix.

The x and y axes are not aligned to Earth coordinate system since magnetometer data are always disturbed by metal constructions in experimental environment facilities, which further affects the accuracy of motion tracking. To correct the magnetometer offset, sensitivity and axis-misalignment, some researches put forward calibration methods [14]. The ellipsoid fitting based on least square method is adopted in this paper for magnetometer calibration with data recorded on-site the day of the event [18]. The remaining angular velocity and acceleration signals are also filtered using second-order digital filter with cut-off frequency at 100 Hz [19]. At the end of signal preprocessing of magnetometer, accelerometer and gyroscope, the data fusion algorithm is used to estimate accurate pose of all human body segment.

### 3.3. Motion Reconstruction Based on Quaternion

Taking into account the gimbal locking problem of Euler angle and its computational complexity of the rotation matrix, the final posture parameters are obtained by fusing acceleration, angular velocity and magnetometer measurement data with the quaternion-based interpretation of body segments rotations [20]. The quaternion is defined by Equation (1).

(1) $$q=q_{0}+q_{1}i+q_{2}j+q_{3}k$$

with the three imaginary units i,j,k, which satisfy the equation $i^{2}=j^{2}=k^{2}=ijk=-1$ [21]. In the initial state, the athlete is asked to face north and stand with his arms down for a few seconds, and the sensor nodes are fixed with a belt on the surface of the body. The initial Euler angles of each sensors nodes are obtained by using Equations (2)–(6). Herein, ϕ, θ and φ, respectively, represent pitch, roll and yaw angle. $a_{x}$, $a_{y}$ and $a_{z}$ are the linear acceleration of the device in three directions, while $m_{x}^{b}$ and $m_{y}^{b}$ are the local magnetic intensity around the test sites after calibration. The rotation quaternion ${}_{s}^{e}q\left(0\right)$ between SCS and ECS at the initial state is also obtained by using Equation (7) [22].

(2) $$\phi =\mathrm{asin}\frac{a_{y}}{\sqrt[{2}]{a_{x}^{2}+a_{y}^{2}+a_{z}^{2}}}$$

(3) $$\theta =\mathrm{atan}2\frac{-a_{x}}{a_{z}}$$

(4) $$m_{x}^{a}=m_{x}^{b}cos\left(\theta \right)+m_{z}^{b}sin\left(\theta \right)$$

(5) $$m_{y}^{a}=m_{x}^{b}sin\left(\theta \right)sin\left(\phi \right)+m_{y}^{b}cos\left(\phi \right)-m_{z}^{b}*cos\left(\theta \right)sin\left(\phi \right)$$

(6) $$\varphi =\mathrm{atan}2\left(m_{y}^{a},m_{x}^{a}\right)$$

(7) $${}_{s}^{e}q\left(0\right)=\left[\begin{array}{c} \cos\left(\frac{\phi }{2}\right)\cos\left(\frac{\theta }{2}\right)\cos\left(\frac{\varphi }{2}\right)-\sin\left(\frac{\phi }{2}\right)\sin\left(\frac{\theta }{2}\right)\sin\left(\frac{\varphi }{2}\right)\\ \cos\left(\frac{\phi }{2}\right)\cos\left(\frac{\theta }{2}\right)\sin\left(\frac{\varphi }{2}\right)-\cos\left(\frac{\phi }{2}\right)\sin\left(\frac{\theta }{2}\right)\sin\left(\frac{\varphi }{2}\right)\\ \cos\left(\frac{\phi }{2}\right)\cos\left(\frac{\theta }{2}\right)\sin\left(\frac{\varphi }{2}\right)+\cos\left(\frac{\phi }{2}\right)\sin\left(\frac{\theta }{2}\right)\sin\left(\frac{\varphi }{2}\right)\\ \cos\left(\frac{\phi }{2}\right)\cos\left(\frac{\theta }{2}\right)\sin\left(\frac{\varphi }{2}\right)+\cos\left(\frac{\phi }{2}\right)\sin\left(\frac{\theta }{2}\right)\sin\left(\frac{\varphi }{2}\right) \end{array}\right]$$

In the initial state, the rotation quaternion ${}_{s}^{b}q\left(0\right)$ between SCS and BCS is equal to ${}_{s}^{e}q\left(0\right)$ because the BCS and ECS overlap, and, since the sensors are placed at fixed position, ${}_{s}^{b}q\left(t\right)$ is always equal to ${}_{s}^{e}q\left(0\right)$ throughout the measurement. The main purpose of the project is to analyze the movement of kayak in terms of the Earth coordinate system. The rotation quaternion of each limb from BCS to ECS can be calculated by Equation (8):

(8) $${}_{b}^{e}q\left(t\right){=}_{s}^{e}q\left(t\right){⊗}_{b}^{s}q\left(t\right)$$

where ${}_{b}^{s}q\left(t\right)$ is the conjugate of ${}_{s}^{b}q\left(t\right)$ and ${}_{s}^{e}q\left(t\right)$ is constantly updated with data fusion algorithm over time. In this paper, the bar-shaped human body model conformed to the rigid body model is defined for representing the human pose. The participant’s pelvis in the ECS is set as the initial position; the posture of each segment is obtained by the iteration of the relationship.

**Figure 6:**
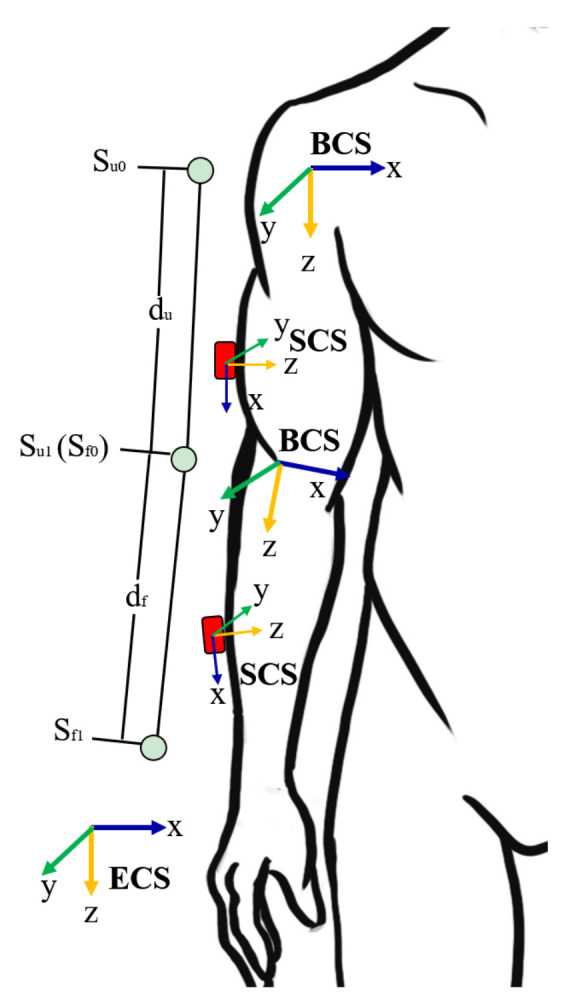
Skeleton structure of upper limb.

Taking the adjacent segment of the upper limb as an example to explain how we iteratively compute the attitude of human body, the upper arm and forearm body segments are modeled as two segments in the elbow joint, as illustrated in Figure 6. $S_{u1}\left(t\right)$ and $S_{f1}\left(t\right)$ are the end position of two segments, while $S_{u0}\left(t\right)$ and $S_{f0}\left(t\right)$ are the start position of two segments. The length vectors of the upper arm and forearm are $d_{u}\left(t\right)$ and $d_{f}\left(t\right)$. Thus, the position of each segment is obtained from Equations (9) and (10). When all segments’ postures of the rigid body model are obtained from the relative skeletal segment iteration calculation, the flexion and extension joint angle can also be solved by inverse cosine between two adjacent skeletal segment vectors.

(9) $$S_{u1}\left(t\right)=S_{u0}\left(t\right)+{(}_{b,u}^{e}q\left(t\right))⊗\left[0d_{u}\left(t\right)\right]⊗{{(}_{b,u}^{e}q\left(t\right))}^{*}$$

(10) $$S_{f1}\left(t\right)=S_{f0}\left(t\right)+{(}_{b,f}^{e}q\left(t\right))⊗\left[0d_{f}\left(t\right)\right]⊗{{(}_{b,f}^{e}q\left(t\right))}^{*}$$

### 3.4. Data Fusion Algorithm

There are many data fusion algorithms for reconstructing human motion, such as complementary filter (CF) [23], gradient descent method (GD) [24] and extended Kalman filter (EKF) [25]. In this article, the EKF method is used for multi-sensor data fusion. The EKF model adopted in this paper is depicted in Equations (11) and (12).

(11) x(t)=f(x(t−1),u,t−1)+w(t−1)

(12) z(t)=h(x(t),t)+v(t)

where x(t) stands for state vector at time *t*; z(t) represents the observation vector at time *t*; *u* indicates the measured values of gyroscope; w(t) and v(t) are the process noise of the state variable and system measured noise; and Q(t) and R(t) denote their covariance matrices, respectively. The state variables are defined as follows, where $q={\left[\begin{array}{cccc} q_{0} & q_{1} & q_{2} & q_{3} \end{array}\right]}^{T}$ represents the pose quaternions and $b_{\omega }={\left[\begin{array}{ccc} b_{\omega x} & b_{\omega v} & b_{\omega z} \end{array}\right]}^{T}$ are the measurements biases of the gyroscope. The state variable vector is written in Equation (13).

(13) $$X={\left[\begin{array}{ccccccc} q_{0} & q_{1} & q_{2} & q_{3} & b_{\omega x} & b_{\omega y} & b_{\omega z} \end{array}\right]}^{T}$$

The updated pose quaternions can be solved by differential equations, which are expressed as Equations (14) and (15).

(14) $$\dot{q}=\frac{1}{2}\hat{q}⊗\omega$$

(15) $$w=\left[\begin{array}{cccc} 0 & -w_{x} & -w_{y} & -w_{z}\\ w_{x} & 0 & w_{z} & -w_{y}\\ w_{y} & -w_{z} & 0 & w_{x}\\ w_{z} & w_{y} & -w_{x} & 0 \end{array}\right]$$

Furthermore, the state transition matrix is obtained by Equations (16)–(18), where *T* denotes the sample periods.

(16) $$F_{q}^{q}\left(t|t-1\right)=\left[\begin{array}{cccc} 0 & -\frac{1}{2}\left(\omega_{x}-b_{\omega x}\right)T & -\frac{1}{2}\left(\omega_{y}-b_{\omega y}\right)T & -\frac{1}{2}\left(\omega_{z}-b_{\omega z}\right)T\\ \frac{1}{2}\left(\omega_{x}-b_{\omega x}\right)T & 0 & \frac{1}{2}\left(\omega_{z}-b_{\omega z}\right)T & -\frac{1}{2}\left(\omega_{y}-b_{\omega y}\right)T\\ \frac{1}{2}\left(\omega_{y}-b_{\omega y}\right)T & -\frac{1}{2}\left(\omega_{z}-b_{\omega z}\right)T & 0 & \frac{1}{2}\left(\omega_{x}-b_{\omega x}\right)T\\ \frac{1}{2}\left(\omega_{z}-b_{\omega z}\right)T & \frac{1}{2}\left(\omega_{y}-b_{\omega y}\right)T & \frac{1}{2}\left(\omega_{x}-b_{\omega x}\right)T & 0 \end{array}\right]$$

(17) $$F_{\omega }^{q}\left(t|t-1\right)=\left[\begin{array}{ccc} \frac{1}{2}q_{1}T & \frac{1}{2}q_{2}T & \frac{1}{2}q_{3}T\\ -\frac{1}{2}q_{0}T & \frac{1}{2}q_{3}T & -\frac{1}{2}q_{2}T\\ -\frac{1}{2}q_{3}T & -\frac{1}{2}q_{0}T & \frac{1}{2}q_{1}T\\ \frac{1}{2}q_{2}T & -\frac{1}{2}q_{1}T & \frac{1}{2}q_{0}T \end{array}\right]$$

(18) $$F\left(t|t-1\right)=\left[\begin{array}{cc} F_{q}^{q} & F_{\omega }^{q}\\ O_{3\times 4} & O_{3\times 3} \end{array}\right]$$

Then, the state transition function is obtained after model linearizing, as shown in Equation (19),

(19) $$\Phi \left(t|t-1\right)=I_{7\times 7}+F\left(t|t-1\right)\times T.$$

Here, let $W\left(t\right)={\left[W_{\omega }\left(t\right),W_{\mathrm{bias}}\left(t\right)\right]}^{T}$ be the vector composed of the noise of gyroscope and the gyroscope bias migration noise. We then proceed to obtain the expressions of the derivatives of two variables. We get $Q\left(t\right)=W\left(t-1\right)\times T\times Q_{w}\times T\times W^{T}\left(t-1\right)$. The covariance matrix $Q_{w}$ is defined in Equation (20).

(20) $$Q_{w}=\left[\begin{array}{cc} I_{3\times 3}\sigma_{\omega } & O_{3\times 3}\\ O_{3\times 3} & I_{3\times 3}\sigma_{\omega b} \end{array}\right]$$

where $\sigma_{\omega }$ and $\sigma_{\omega b}$ are the initial value of bias and migration noise of gyroscope. Then, the prior state estimate can be calculated from state transition function (Equation (21)):

(21) $$P\left(t|t-1\right)=\Phi \left(t|t-1\right)P\left(t-1|t-1\right)\Phi^{T}\left(t|t-1\right)+Q\left(t\right)$$

In this paper, the acceleration vector and magnetic field vector are selected as the observation variables, which are defined as Equation (22), where $a_{x}$, $a_{y}$ and $a_{z}$ are the measurements of three-axis accelerations and $m_{x}$, $m_{y}$ and $m_{z}$ represent the measurements of three-axis magnetometer on the horizontal plane.

(22) $$Z={\left[\begin{array}{cccccc} a_{x} & a_{y} & a_{z} & m_{x} & m_{y} & m_{z} \end{array}\right]}^{T}$$

The observation matrix of the accelerometer $H_{a}$ is calculated on the projection of the gravitation on the carrier coordinate system by Equation (23).

(23) $$H_{a}{=}_{e}^{b}R\left[\begin{array}{c} 0\\ 0\\ -g \end{array}\right]=\left[\begin{array}{c} -2g\left(q_{1}q_{3}-q_{0}q_{2}\right)\\ -2g\left(q_{2}q_{3}+q_{0}q_{1}\right)\\ -g\left(q_{0}^{2}-q_{1}^{2}-q_{2}^{2}+q_{3}^{2}\right) \end{array}\right]$$

where ${}_{e}^{b}R$ indicates the rotation matrix between SCS and ECS. The observation matrix $H_{mag}$ is computed in the same fashion as $H_{a}$, and the magnetic field values ${\left[\begin{array}{ccc} h_{x} & 0 & h_{z} \end{array}\right]}^{T}$ also need to be projected into the carrier coordinate system. The observation matrix *H* is calculated by Equation (24).

(24) $$H=\left[\begin{array}{cc} H_{\mathrm{mag}} & 0_{3\times 3}\\ H_{a} & 0_{3\times 3} \end{array}\right]$$

Therefore, the state gain matrix can be expressed as Equation (25).

(25) $$K\left(t\right)=P\left(t|t-1\right)H^{T}\left(t\right){\left[H\left(t\right)P\left(t|t-1\right)H^{T}\left(t\right)+R\left(t\right)\right]}^{-1}$$

Thus, the estimate value of the state vector at time *t* can be calculated from Equation (26), and the state error covariance matrix is updated by Equation (26). K(t) represents the gain factor, X(t∣t) is the posteriori state estimate and P(t) is the posterior covariance matrix, as shown in Equation (27).

(26) X(t∣t)=X(t−1∣t)+K(t)[Z(t)−h(X(t∣t−1))]

(27) P(t)=[I−K(t)H(t)]P(t∣t−1)

### 3.5. Evaluation Method for Sprint Kayak Technique

The kayakers under different competitive levels vary substantially in trunk rotation, leg motion, stroke width, stroke rate, overall motion of the kayak, blade and water-contact time and other factors [26]. From these important factors, the most influential is the stroke frequency, stroke phases and stroke variation. After motion reconstruction, the details about the action implementation are also captured despite the visual occlusion conditions. The joint angles were estimated by computing the reverse cosine of the angle between the adjacent segment vectors. Then, the motion information is retained for further analysis.

**Figure 7:**
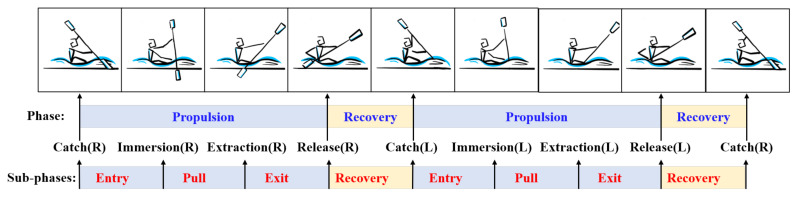
The model for kayak motion analysis including two levels: phases and sub-phases. The phases defining positions are entry, pull, exit and return. R, right side; L, left side.

There are many parametric ways to estimate the technical level of athlete based on the joint angles series. To achieve the best performance, the efficient and consistent stroke cycle is deemed necessary. The information about stroke rhythm can be calculated by searching the joint angle signal peak values. The consistency and variation are also identified by the movement range of joint at the extremities. In addition to these, the stroke phase is a more practical indicator used by coaches. A stroke cycle is defined as the periodic movement. When analyzing rowing technique, the stroke cycle is usually broken down into 2–4 phases, and there are many observational models to distinguish movement phases. In this paper, the model adopted for the categorization and analysis of kayaking movement is shown in Figure 7. The first-level phase is defined as the period of the whole stroke cycle, which corresponds to before and after the same-side paddle blade enteris the water. For detailed division of the stroke cycle, the propulsion phase is separated based on water contact of the paddle, which is considered for the greater visibility of the position. The four sub-phases are defined by the instant of the blade catching, immersing, extracting and releasing from the water. The sub-phases are divided into paddle entry, pull phase, paddle exit and recovery phase within a larger phase. The duration of pull phase and the ratio of propulsion duration to recovery duration have significant effects on the rowing performance [27].

### 3.6. Feature Extraction Method

To make predictions on the joint angle sequence data based on the phase partitioning method described above, the present study proposes several machine learning models to predict the movement phases (entry, pull, exit and recovery) based on the feature matrix of upper limbs joint angles. Statistical descriptive feature extraction is a widely used method to calculate the statistical features on the sample record [28]. However, the duration, amplitude and orientation vary among athletes. To obtain more comprehensive information for further phase recognition, the wavelet scattering transform is proposed in this paper for feature extraction [29]. Its algorithm principle is as follows.

The input signal $x=\left[x_{1},x_{2},\ldots ,x_{n}\right]$ is a n-dimensional vector whose length is the number of joint angle series under analysis. $\psi_{(}x)$ is the chosen wavelet mother function, which is used for multiscale and oriented filter bank. The definition is described as follows.

(28) $$\psi_{\lambda_{i}}\left(x\right)=2^{-2j_{i}}\psi \left(\lambda_{i}x\right)$$

where θ∈R represents the rotation matrix in the finite, discrete rotation group *R* and $\lambda_{i}=2^{-j_{i}}\theta_{i},i=1,\ldots ,m$ represents the joint scaling and rotation operators. ϕ(x) is denoted as the low-pass filter, and its definitions is described as follows.

(29) $$\phi_{J}\left(x\right)=2^{-2J}\phi \left(2^{-J}x\right)$$

In the rest of this paper, *j* and *J* are integers within j≤J, and *j* is the level of scattering. The wavelet scattering transform has important advantages of invariant, stable and informative signal feature representation. The following methods is used to recover the information by the operation of the invariant modulus part. We denote $S_{i}x,i=1,2,\ldots ,m$ as the wavelet scattering coefficients of each layer. The output of each layer is written by the function of the modulus, and the low-pass averaging function is described as follows:

(30) $$S_{0}x=x*\phi_{J}\left(x\right)$$

(31) $$S_{1}x\left(x,\lambda \right)=\left|x*\psi_{\lambda }\right|*\phi_{J}\left(x\right)$$

(32) $$S_{m}x\left(x,\lambda_{1},\ldots ,\lambda_{m}\right)=\left|\ldots \right|x*\psi_{\lambda_{1}}\left|\ldots *\psi_{\lambda_{m}}\right|*\phi_{J}\left(x\right)$$

The final wavelet scattering coefficients are the whole output of the transform from the 0th to mth order, as expressed in Equation (33).

(33) $$Sx=\left\{S_{0}x,S_{1}x,\ldots S_{m}x\right\}$$

When the scattering transform of all slide-windowed joint angle record are obtained, the feature matrixes based on wavelet scattering transform are fed into machine learning model for training and predicting movement phases.

## 4. Results

### 4.1. Performance Comparison between Self-Made System and Standard Optical System

**Figure 8:**
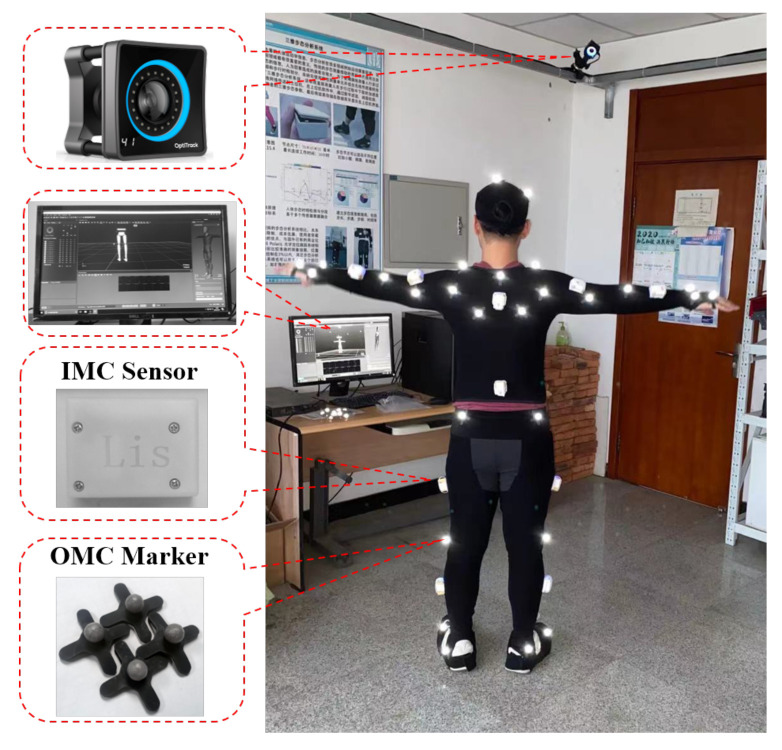
Inertial motion capture (IMC) sensor and optical motion capture (OMC) marker setup shown in the room.

To verify the performance of the self-made inertial motion capture system (IMC), we set a contrast experiment with the commercial optical motion capture system (OMC). The OMC is mainly considered as the reference standard tool for dynamic measurement of upper and lower limb joint angles. The experimental arrangement is schematically shown in Figure 8. The participant was instructed to wear inertial sensor nodes and the markers of OMC simultaneously and make stretching exercises of the trunk and extremities. Due to the limitations of OMC, the participants wore tight-fighting shorts for the data collection to avoid occlusions. The whole-body movements were recorded using the 3D optical motion capture system (OptiTrack, American) and inertial sensor nodes. The experiment were approved by The University of Dalian Technology at LiaoNing province China and all participants provided written informed consent.

**Figure 9:**
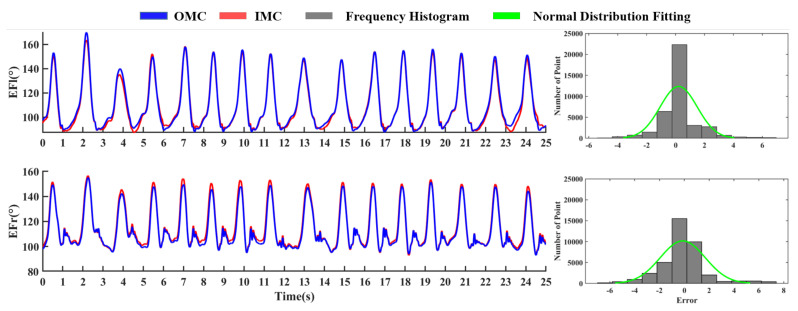
Contrast curve of elbow flexion and extension angle and error statistics.

The coordinate system between the OMC and IMC is inconsistent, thus the raw data of OMC need to be converted. The results of comparison between OMC and IMC are presented as follows. Figure 9 shows a comparison graph of the elbow flexion extension for the two systems. First, consider the situation in the left half of Figure 9. The IMC can trace the motion curve accurately compared to OMC. The corresponding correlation coefficients are 0.995 and 0.996 of the two curves, respectively. The measured values of the optical system are used as a standard reference. The descriptive statistical histograms of the relative errors are also calculated for the right half of Figure 9, and the measurement errors are well controlled. All results show that the device we developed is reliable [30].

### 4.2. Kinematic Statistical Analysis after Motion Reconstruction

**Figure 10:**
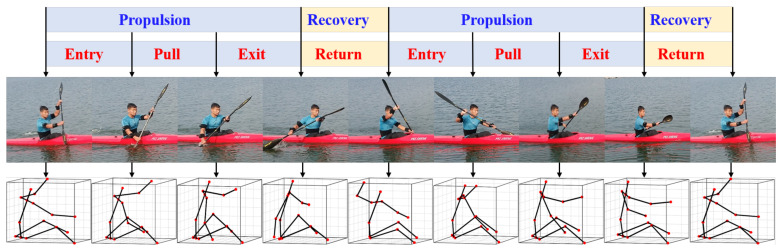
The result of motion reconstruction using the proposed method.

The kayaker sprint involves the motion of the whole body. The paddling movement involves arm and trunk muscles, but it needs to activate the bilateral extensors and flexors of the hips and knees to simultaneously twist the body. Only in this way, the power output will be increased, and a higher velocity is obtained. Thus, the motion of whole body was captured in our experiments. All experiments were repeated at least three times for lengths of 200 m each time. At the same time, the entire process as recorded with a motion camera from coronal plane at 240 frame rate. Figure 10 shows the results of motion reconstruction using IMC equipment. To guarantee safe conditions, the observation of camera-based motion capture maintained constant viewing distance. It is inevitable that the vision of lower extremities is occluded by the hull and sometimes the upper limbs are occluded by the paddle. However, the details of motion expression are captured by the IMC, which is proven to be an efficient method to overcome the difficulty caused by the visual method [24].

**Figure 11:**
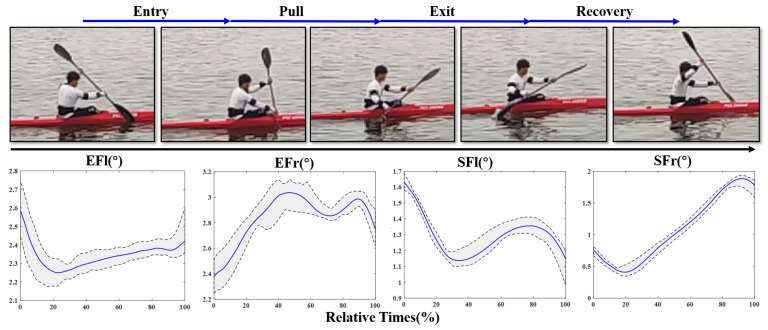
Joint angle transform curves of elbow and shoulder during right-side stroke cycle.

**Figure 12:**
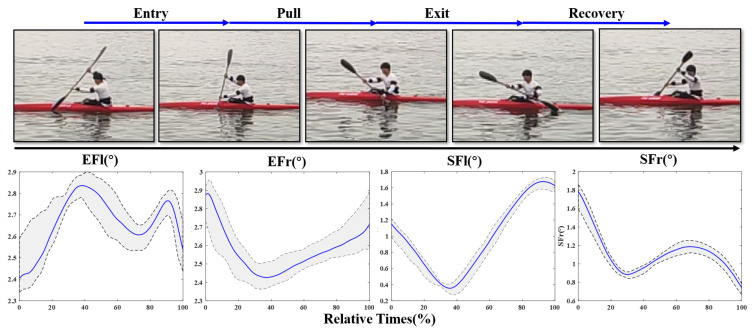
Joint angle transform curves of elbow and shoulder during left-side stroke cycle.

The elbow and shoulder joint angle curves of a stroke cycle are shown in Figure 11 and Figure 12, where the blue solid lines represent the time-normalized group mean and the black dotted lines represent the time-normalized maximum (MAX) and minimum mean (MIN). The gray shaded area represents the range of motion (ROM) between MAX and MIN. The mean value and standard deviation of MAX, MIN and ROM of elbow and shoulders are also calculated, and the results are shown in Table 1. A stroke cycle is completed in two stages of right-side and left-side stroke. During the two sub-phases, the paddlers try to maintain similar extremity rotation and flexion angles. It can be seen in the graph that joint angles of elbow and shoulder in right-side stroke is the opposite of the extremities movements in left-side stroke. This also accounts for the similarity between EFl in Figure 11 and EFr in Figure 12, and the same applies for EFr, SFr and SFl.

During the stroke, the draw elbow is extended, while the draw shoulder is flexed in an attempt to place the blade paddle in the water as far as toward the boat as possible. To maintain the optimal stroke performance, the kayakers alter shoulder and arm musculature to resist the external forces. Because the upper limbs movements accounted for approximately half of the stroke cycle, shoulder injuries are always observed in elite kayakers [31]. Besides the kinematic asymmetries of the whole-body during paddling, the MAX, MIN and ROM of elbow and shoulder flexion are important reasons for muscle strain [32]. As shown in Figure 11 and Figure 12 and Table 2, the MEAN, MAX and MIN values of elbow are larger than those of shoulder. However, the ROM of shoulder is much larger than that of elbow. The maximal shoulder angle of dominant limb appears at around 80% into the cycle. The peak of shoulder extension occurs during the abductor movement of the recovery phase in a stroke cycle. These observed results are similar to previous studies [31,33]. This illustrates that the power output for rowing is mainly completed by the upper arm and shoulder muscles. According to the above analysis, the potential for shoulder and scapular injury is monitored and avoided as much as possible.

The proficient stroke would ideally be symmetrical on both sides in order to propel the kayak straight and distribute forces equally on the body. The elite kayakers always use a similar, consistent rowing technique. The body motion and posture do not change during the whole process. In this study, all athletes could be regard as junior rowers. Based on the time-normalized group joint angles series, the correlations on opposite curves of both sides are 0.8045, 0.7607, 0.9326 and 0.8548, respectively. These parameters represent the degree of proficiency, which is the kinematic difference among elite, junior and beginner kayakers, where elites have a perfect pattern of symmetry between the left to right, which are also the inadequacies for beginners to improve themselves.

Obviously, this above kinematic statistical analysis is comprehensible and intuitive, and it can assist coaches to draw up the training program and avoid sport injury. Different from the in-house analysis based on the training equipment, the real on-water movement analysis is more responsive and makes the results more pertinent.

### 4.3. Phase Partition Based on the Joint Angles Series

Phases are always described in the stroke quality analysis [34]. The two-phase model including propulsion and recovery phase is a widely used indicator. For more detailed analysis on the premise of not disrupting the two-phase model, the sub-phase model including entry, pull, exit and return can be used. In this study, to predict the phase of motion process automatically, machine learning algorithms were used to classify the phase based on the wavelet scattering feature matrix of four joint angles series, and the durations of each phase were obtained.

High speed motion camera was used to capture the kayaker’s movement at the recording rate of 240 frames per second, and, then, the four joint angle series were annotated with video images as the comparator groups. Next, the sliding window of 20 data points with 50% overlap was applied for the different phase series. We ended up with a dataset of 11,851 pieces of labeled sequences. Because the energy of wavelet scattering decreases rapidly as the layer level increases, and almost 99% of the energy is contained in the first two layers, we focused on the first two layer scattering coefficients [35], and then the features extracted from the labeled records using wavelet scattering were combined to predict the class membership of data. Movement recognition consists of identifying special actions and the stage at which the object is currently at. Fine-grained movement recognition is focuses on distinguishing different actions between subtle changes. We try to propose sensor-based recognition and evaluation models based on joint angle series. Because the number of scattering features is large, several simple classifiers including Support Vector Machine (SVM), Logistic Regression, Decision Tree, K-Nearest Neighbor (KNN) and Random Forest were considered, and we compared their performance. The whole feature dataset was divided into training (80%) and test sets (20%). The training dataset as used to train the models and the remaining dataset was used for predicting. Standard measurements were used to evaluate the performance of each selected model: classification accuracy, precision, recall and F1-score. The receiver operating characteristic curve (ROC) was obtained from the false positive rate against the true positive rate under the different thresholds. The precision–recall curve (PRC) was derived from the relationship between precision and recall with the different thresholds. The corresponding areas under the curves (AUC) of ROC and PRC were also calculated to assess the prediction performance. The model with a higher area (between 0 and 1) value gives a better predictive performance. The specific details for recognition performance are listed in Table 3.

The trained model was used to predict the phase position of the new motion sequences collected from the participants. Next, we calculated the duration of each sub-phase of all participants. The result is shown in Table 4. As described in a previous paper [36], experienced kayakers have lower standard deviation (SD) values than novices, and SD can be regarded as an indicator of the joint angle consistency of the kayakers. Table 4 shows that the third and fourth athletes present relatively small standard deviations. The results reflect that they have achieved better performance over the experiment time.

**Figure 13:**
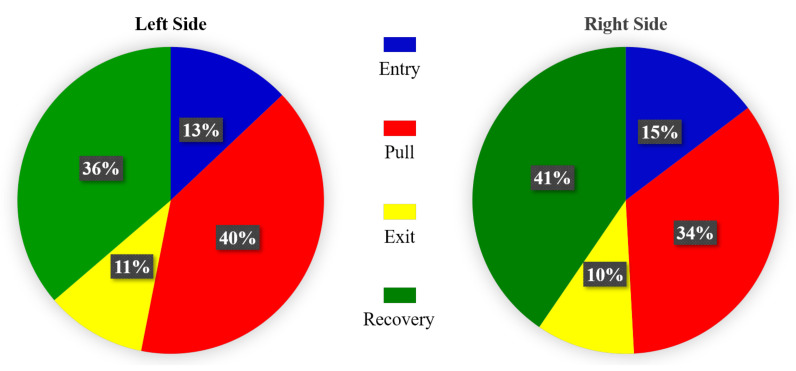
The ratio of different phase duration to total time in a stroke cycle.

The duration ratio of different phase on both sides towards all participant was also calculated, as plotted in Figure 13. The acceleration procedure of kayak is mainly completed during the propulsion phase. More precisely, the entry and pull phase complete most of the work. As shown in Figure 13, the ratio between propulsion and recovery phase of all experimenters in this study is close to 60%. For many elite athletes, efforts are made to minimize the duration of recovery process and increase the duration of the propulsion phase, and this is more effective than enhancing the stroke rate for increasing the boat velocity [37,38]. On the other hand, it can be seen that the symmetry between left side and right side is imperfect. Asymmetry has been related to injuries in kayakers during the paddle stroke [33].

By the above quantitative analysis of the stroke cycle, the stroke quality of kayakers can be evaluated from different perspectives. The coach will be able to provide specific recommendations to the athletes, and the performance of kayakers can be improved.

## 5. Discussion

The whole-body posture is important to the paddling technique [39], and rowing technique is largely composed of posture and timing. This work tries to explore these aspects further. We used inertial sensors to acquire kayakers’ motion kinematic information during rowing, which is powerful and permits more precise and specific correlations between performance and proficient level. All sequence data of whole-body activity obtained in this study are available under real on-water environment. The experimental results, including duration of stroke cycle, stroke frequency, ROM of limbs movement, similarity of both sides, rate of propulsion/recovery phase, etc. are consistent with previous study [2,3,31,36,40].

Although the satisfactory results are also seen in the comparison and prediction of the follow-up experimental arrangement, indeed, the performance of the athletes could be influenced by many factors. The distinct differences are also show in only short time period even for the same individual. Consequently, the timeliness of provided information obtained by the proposed method is important for communication between coaches and athletes. On the other hand, wearing multiple sensors have resulted in bodily discomfort and further it would affect the coordination between the limbs and the spine. Therefore, it is necessary to minimize the number of wearable devices or significantly decrease the size and weight of sensors nodes. Besides, we next set out to optimize the rigid model, including decreasing the number of degrees of freedom, and so on. After adjusting the above possible factors, a more comprehensive water sport athlete monitoring system will be established in the future.

## 6. Conclusions

In this work, we present a systematic method for athlete’s motion capture and kinematic analysis. The customizable rigid model is used to demonstrate the kayaker’s posture, and each segment attitude of the whole-body is iteratively calculated by the quaternization vector multiplication. The contrast test indicates that the proposed approach has comparable accuracy to the standard commercial optical motion capture system. This paper highlights the range of motion of the extremities, which is essential for preventing sports injury, and the duration of motion phases, which is the import index for competitive level. The detailed kinematic analysis based on the field on-water experiments could be provided, and this enable coaches to give targeted feedback and guidance based on the participant’s activities in real scenario.

## Figures and Tables

**Table 1:** Sensor detailed Specifications.

Unit	Accelerometer	Gyroscope	Magnetometer
Dimensions	3 axis	3 axis	3 axis
Sensitivity(/LSB)	0.833 mg	0.04 deg/s	142.9 uguass
Dynamic Range	±18 g	±1200 deg/s	±1.9 gauss
−3 dB Bandwidth(Hz)	330	330	25
Nonlinearity(%FS)	0.2	±0.1	0.1
Misalignment(deg)	0.2	0.05	0.25

**Table 2:** Summary statistics of joint angle sequencing data.

MEAN ± SD (deg)		ROM	MAX	MIN	MEAN
Left Side	EFl	21.9 ± 3.1	167.0 ± 2.1	145.2 ± 1.6	156.4 ± 1.5
	EFr	37.6 ± 6.4	158.3 ± 5.9	120.6 ± 1.6	129.0 ± 1.6
	SFl	74.1 ± 2.0	99.9 ± 1.9	25.8 ± 1.6	58.9 ± 1.3
	SFr	60.2 ± 4.5	103.1 ± 3.4	41.0 ± 1.7	63.9 ± 2.0
Right Side	EFl	20.3 ± 1.9	147.7 ± 3.8	127.5 ± 1.9	132.5 ± 1.9
	EFr	36.9 ± 4.9	174.9 ± 3.4	138.0 ± 2.9	155.8 ± 2.2
	SFl	29.6 ± 2.4	94.6 ± 2.3	65.0 ± 1.4	75.5 ± 1.4
	SFr	88.6 ± 2.6	110.0 ± 2.0	21.5 ± 2.2	62.3 ± 1.5

**Table 3:** Prediction performance comparison.

Evaluation Metrics	Machine Learning Algorithm				
	Decision Tree	KNN	SVM	Logistic Regression	Random Forest
Accuracy	**0.9827**	**0.9898**	**0.9898**	**0.9789**	**0.9856**
Precision	0.9826	0.9900	0.9898	0.9787	0.9856
Recall	0.9827	0.9899	0.9899	0.9789	0.9857
F1-score	0.9826	0.9899	0.9898	0.9788	0.9855
AUC of ROC	0.9834	0.9993	0.9999	0.9990	0.9992
AUC of PRC	0.9679	0.9984	0.9996	0.9976	0.9981

**Table 4:** The duration of sub-phases during one round trip.

MEAN ± SD (ms)	Left Side				Right Side			
	Entry	Pull	Exit	Recovery	Entry	Pull	Exit	Recovery
Subject 1	124 ± 13	396 ± 28	116 ± 15	388 ± 22	144 ± 15	299 ± 23	95 ± 16	371 ± 28
Subject 2	120 ± 14	414 ± 14	118 ± 17	385 ± 24	152 ± 14	301 ± 22	100 ± 14	385 ± 32
Subject 3	121 ± 6	354 ± 19	86 ± 12	319 ± 18	129 ± 10	297 ± 16	96 ± 12	337 ± 14
Subject 4	132 ± 8	369 ± 17	96 ± 11	328 ± 22	131 ± 9	300 ± 16	96 ± 9	343 ± 15
Subject 5	131 ± 10	435 ± 32	105 ± 13	370 ± 19	133 ± 7	333 ± 20	101 ± 12	405 ± 22
Subject 6	127 ± 9	428 ± 27	103 ± 14	361 ± 21	127 ± 9	340 ± 23	91 ± 18	393 ± 26

## Abbreviations

The following abbreviations are used in this manuscript:

IMUs	Inertial Measurement Units
ECS	Earth Coordinate System
SCS	Sensor Coordinate System
BCS	Body Coordinate System
SF	Shoulder Flexion Angle
EF	Elbow Flexion Angle
KF	Knee flexion Angle
FF	Foot Flexion Angle
SFl	Left Shoulder Flexion Angle
SFr	Right Shoulder Flexion Angle
EFl	Left Elbow Flexion Angle
EFr	Right Elbow Flexion Angle
CF	Complementary Filter
GD	Gradient Descent
EKF	Externed Kalman Filter
IMC	Inertial Motion Capture System
OMC	Optical Motion Capture System
SVM	Support Vector Machine
KNN	K-Nearest Neighbor
ROM	Range of Motion
MAX	Maximum Value
MIN	Minimum Value
MEAN	Mean Value
SD	Standard Deviation
ROC	Receiver Operating Characteristic
PRC	Precision Recall Curve
AUC	Area Under the Curve

## References

[1] Borges T.O., Bullock N., Duff C., Coutts A.J. (2014). Methods for quantifying training in sprint kayak. J. Strength Cond. Res..

[2] Lee C.H., Nam K.J. (2012). Analysis of the kayak forward stroke according to skill level and knee flexion angle. Int. J. Bio-Sci. Bio-Technol..

[3] Brown B.M., Lauder M., Dyson R. (2011). Notational analysis of sprint kayaking: Differentiating between ability levels. Int. J. Perform. Anal. Sport.

[4] Tay C.S., Kong P.W. (2018). A Video-based Method to Quantify Stroke Synchronisation in Crew Boat Sprint Kayaking. J. Hum. Kinet..

[5] Gomes B.B., Ramos N.V., Conceiç ao F.A., Sanders R.H., Vaz M.A., Vilas-Boas J.P. (2015). Paddling force profiles at different stroke rates in elite sprint kayaking. J. Appl. Biomech..

[6] Gravenhorst F., Tessendorf B., Adelsberger R., Arnrich B., Tröster G., Thiem C. (2012). SonicSeat: A seat position tracker based on ultrasonic sound measurements for rowing technique analysis. BODYNETS.

[7] Henry J.C., Clark R.R., McCabe R.P., Vanderby R. (1995). An evaluation of instrumented tank rowing for objective assessment of rowing performance. J. Sport. Sci..

[8] Ridge B.R., Broad E., Kerr D.A., Ackland T.R. (2007). Morphological characteristics of Olympic slalom canoe and kayak paddlers. Eur. J. Sport Sci..

[9] Plagenhoef S. (1979). Biomechanical analysis of Olympic flatwater kayaking and canoeing. Res. Quarterly. Am. Alliance Heal. Phys. Educ. Recreat. Danc..

[10] Sturm D., Yousaf K., Eriksson M. A wireless, unobtrusive kayak sensor network enabling feedback solutions. Proceedings of the 2010 International Conference on Body Sensor Networks.

[11] Qiu S., Wang Z., Zhao H., Hu H. (2016). Using distributed wearable sensors to measure and evaluate human lower limb motions. IEEE Trans. Instrum. Meas..

[12] Tesconi M., Tognetti A., Scilingo E.P., Zupone G., Carbonaro N., De Rossi D., Castellini E., Marella M. Wearable sensorized system for analyzing the lower limb movement during rowing activity. Proceedings of the 2007 IEEE International Symposium on Industrial Electronics.

[13] King R.C., McIlwraith D.G., Lo B., Pansiot J., McGregor A.H., Yang G.Z. Body sensor networks for monitoring rowing technique. Proceedings of the 2009 Sixth International Workshop on Wearable and Implantable Body Sensor Networks.

[14] Ruffaldi E., Peppoloni L., Filippeschi A. (2015). Sensor fusion for complex articulated body tracking applied in rowing. Proc. Inst. Mech. Eng. Part P J. Sport. Eng. Technol..

[15] Hamano S., Ochi E., Tsuchiya Y., Muramatsu E., Suzukawa K., Igawa S. (2015). Relationship between performance test and body composition/physical strength characteristic in sprint canoe and kayak paddlers. Open Access J. Sport. Med..

[16] Wang Z., Li J., Wang J., Zhao H., Qiu S., Yang N., Shi X. (2018). Inertial sensor-based analysis of equestrian sports between beginner and professional riders under different horse gaits. IEEE Trans. Instrum. Meas..

[17] McKean M.R., Burkett B. (2010). The relationship between joint range of motion, muscular strength, and race time for sub-elite flat water kayakers. J. Sci. Med. Sport.

[18] Hemerly E.M., Coelho F.A.A. (2014). Explicit Solution for Magnetometer Calibration. IEEE Trans. Instrum. Meas..

[19] Falbriard M., Mohr M., Aminian K. (2020). Hurdle Clearance Detection and Spatiotemporal Analysis in 400 Meters Hurdles Races Using Shoe-Mounted Magnetic and Inertial Sensors. Sensors.

[20] Choukroun D., Bar-Itzhack I.Y., Oshman Y. (2006). Novel quaternion Kalman filter. IEEE Trans. Aerosp. Electron. Syst..

[21] Salchow-Hömmen C., Callies L., Laidig D., Valtin M., Schauer T., Seel T. (2019). A tangible solution for hand motion tracking in clinical applications. Sensors.

[22] Pio R. (1966). Euler angle transformations. IEEE Trans. Autom. Control.

[23] Wu Z., Jiang X., Zhong M., Shen B., Zhang L. (2020). Wearable Sensors Measure Ankle Joint Changes of Patients with Parkinson’s Disease before and after Acute Levodopa Challenge. Park. Dis..

[24] Liu L., Qiu S., Wang Z.L., Li J., Wang J.X. (2020). Canoeing Motion Tracking and Analysis via Multi-Sensors Fusion. Sensors.

[25] Li J., Wang Z., Qiu S., Zhao H., Yang N. (2019). Study on Horse-rider Interaction Based on Body Sensor Network in Competitive Equitation. IEEE Trans. Affect. Comput..

[26] Worsey M.T., Espinosa H.G., Shepherd J.B., Thiel D.V. (2019). A systematic review of performance analysis in rowing using inertial sensors. Electronics.

[27] McDonnell L.K., Hume P.A., Nolte V. (2012). An observational model for biomechanical assessment of sprint kayaking technique. Sport. Biomech..

[28] Wang J., Wang Z., Qiu S., Xu J., Zhao H., Fortino G., Habib M. (2020). A selection framework of sensor combination feature subset for human motion phase segmentation. Inf. Fusion.

[29] Singh A., Kingsbury N. Dual-tree wavelet scattering network with parametric log transformation for object classification. Proceedings of the 2017 IEEE International Conference on Acoustics, Speech and Signal Processing (ICASSP).

[30] Fleron M.K., Ubbesen N.C.H., Battistella F., Dejtiar D.L., Oliveira A.S. (2018). Accuracy between optical and inertial motion capture systems for assessing trunk speed during preferred gait and transition periods. Sport. Biomech..

[31] Fleming N., Donne B., Fletcher D. (2012). Effect of kayak ergometer elastic tension on upper limb EMG activity and 3D kinematics. J. Sport. Sci. Med..

[32] Limonta E., Squadrone R., Rodano R., Marzegan A., Veicsteinas A., Merati G., Sacchi M. (2010). Tridimensional kinematic analysis on a kayaking simulator: Key factors to successful performance. Sport Sci. Health.

[33] Wassinger C.A., Myers J.B., Sell T.C., Oyama S., Rubenstein E.N., Lephart S.M. (2011). Scapulohumeral kinematic assessment of the forward kayak stroke in experienced whitewater kayakers. Sport. Biomech..

[34] McDonnell L.K., Hume P.A., Nolte V. (2013). A deterministic model based on evidence for the associations between kinematic variables and sprint kayak performance. Sport. Biomech..

[35] Bruna J., Mallat S. (2013). Invariant scattering convolution networks. IEEE Trans. Pattern Anal. Mach. Intell..

[36] Bosch S., Shoaib M., Geerlings S., Buit L., Meratnia N., Havinga P. Analysis of indoor rowing motion using wearable inertial sensors. Proceedings of the 10th EAI International Conference on Body Area Networks.

[37] Trevithick B.A., Ginn K.A., Halaki M., Balnave R. (2007). Shoulder muscle recruitment patterns during a kayak stroke performed on a paddling ergometer. J. Electromyogr. Kinesiol..

[38] Černe T., Kamnik R., Vesnicer B., Gros J.Ž., Munih M. (2013). Differences between elite, junior and non-rowers in kinematic and kinetic parameters during ergometer rowing. Hum. Mov. Sci..

[39] Kemecsey I. (1986). Personal communication.

[40] Bjerkefors A., Tarassova O., Rosén J.S., Zakaria P., Arndt A. (2018). Three-dimensional kinematic analysis and power output of elite flat-water kayakers. Sport. Biomech..

